# Supplementary material for: Emerging exotic compositional order on approaching low-temperature equilibrium glasses
Source: Nat Commun. 2023 Aug 7;14:4614. doi: 10.1038/s41467-023-40290-1 (PMC10406820; doi:10.1038/s41467-023-40290-1)
Supplement: Supplementary file 1 — Supplementary Information [file 41467_2023_40290_MOESM1_ESM.pdf]

Supplementary Information for "Emerging exotic compositional order on approaching low-temperature equilibrium glasses"

Hua Tong[1,2] and Hajime Tanaka[2,3]
[1] *Department of Physics, University of Science and Technology of China, Hefei 230026, China*
[2] *Department of Fundamental Engineering, Institute of Industrial Science, The University of Tokyo, 4-6-1 Komaba, Meguro-ku, Tokyo 153-8505, Japan*
[3] *Research Center for Advanced Science and Technology, The University of Tokyo, 4-6-1 Komaba, Meguro-ku, Tokyo 153-8904, Japan*

SUPPLEMENTARY NOTE 1. STATIC STRUCTURE FACTOR

Corresponding to Fig. 2d in the main text, Fig. 1 shows the static structure factor $S(k)$ from high temperatures in the simple liquid state down to the lowest temperature under study $T = 0.03$. No obvious change is observed in $S(k)$, especially in the low-k range below the first main peak at around $k = 2\pi$. Therefore, the exotic compositional order is undercover from the conventional structural characterisations like $S(k)$ [1].

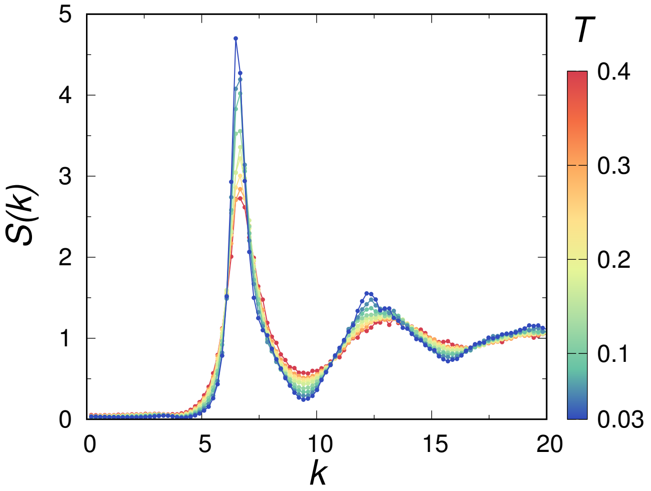

Supplementary Figure 1: **Static structure factor $S(k)$ over a range of temperatures**.

SUPPLEMENTARY NOTE 2. ADDITIONAL ANALYSIS OF STRUCTURE

Here we further characterise the structural evolution as a function of temperature. In the main text, we have categorised the particles into three groups according to whether their coordination number z is less, equal, or more than 6. In Fig. 2a, we show more detailed information on the fraction of particles with $z \in [3, 9]$. It is seen that $z = 5$ and $z = 7$ dominate the groups of $z < 6$ and $z > 6$, respectively, whereas the fractions of particles with $z = 4$ and $z = 8$ are much smaller and further decrease with decreasing temperature. Moreover, we find that small particles tend to have $z = 5$ or $z = 4$, whereas large particles tend to have $z = 7$ or $z = 8$. Therefore, it is reasonable to categorise the particles into three groups ($z <, =, \text{and} > 6$) without more detailed differentiation.

Figure 2b shows the temperature dependence of the average diameter for particles with different coordination numbers z. Clearly, particles with $z < 6$, $z = 6$, and $z > 6$ have a close correspondence with those of small, medium, and large sizes. The weak temperature dependence of average diameter also suggests that such a tendency holds in the studied temperature range.

Furthermore, Fig. 2c shows the temperature dependence of the average Ψ_6 of particles with different coordination numbers z. Overall, the average Ψ_6 for $z = 6$ particles increases with decreasing temperature because of the optimisation of hexatic order under reduced thermal fluctuations. On the contrary, optimising other folds of rotational

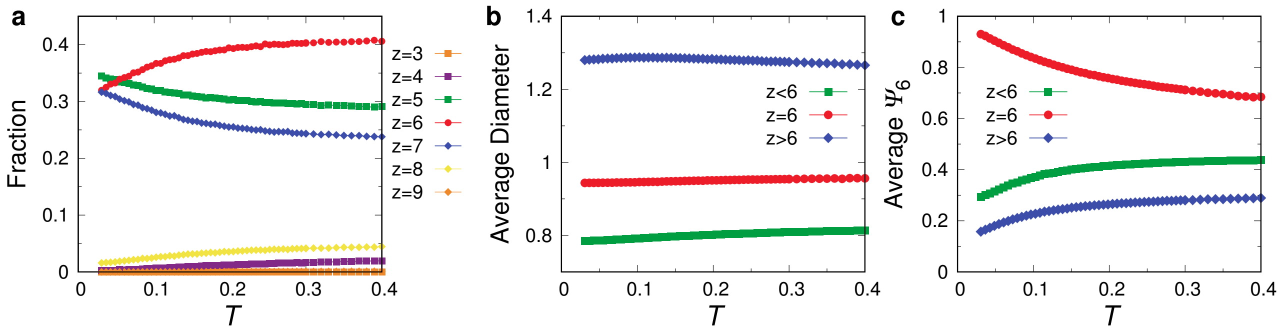

Supplementary Figure 2: **Temperature dependence of several structural properties**. Temperature dependence of the fraction of particles with different coordination numbers z (**a**), the average diameter of particles with different coordination numbers z (**b**), and the average Ψ_6 of particles with different coordination numbers z (**c**).

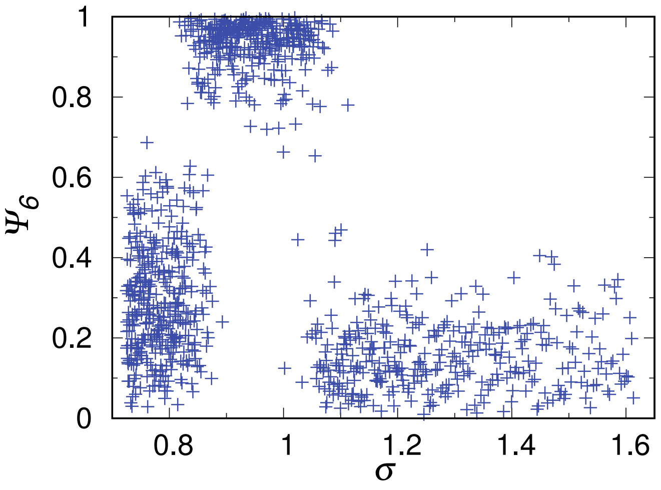

Supplementary Figure 3: **Relationship between the particle size and hexatic order parameter**. A scatter plot of particle diameter σ verse Ψ_6 for a typical configuration at $T = 0.03$, which reveals three major species of particles with different sizes.

symmetry decreases the average Ψ_6. Together with the results of the fraction of particles with different z (see Fig. 2b of the main text), this gives rise to the peak of $\overline{\Psi}_6(T)$ shown in Fig. 2a of the main text.

To better illustrate the relationship between the particle size and hexatic order parameter in more detail, in Fig. 3, we show a scatter plot of particle diameter σ versus Ψ_6 for a typical configuration at $T = 0.03$. Three species of particles with the smallest, medium, and largest sizes are characterised by different values of Ψ_6. Overall, small (large) particles tend to have $z < 6$ ($z > 6$) and, therefore, low values of Ψ_6, whereas particles with medium sizes tend to have $z = 6$ and, therefore, high values of Ψ_6. This result supports the picture of exotic compositional order with clearly different components contributed by particles with different sizes.

It is well-known that particles may tend to be surrounded by neighbours with particular characters in simple mixtures or polydisperse systems. This phenomenon is referred to as "compositional order", which reflects the local structure ordering. For example, in simple binary mixtures of repulsive particles, large and small particles tend to phase-separate, whereas, in the Kob-Anderson model with nonadditive interactions, large and small particles tend to stay together. Here we check how the nonconventional structure ordering is reflected in the usual compositional-order characterisation. Following Ref. 2, we consider the fluctuation in the diameter of neighbouring particles. The average neighbour diameter of particle i is defined as

$$\tilde{\sigma}(i) = \frac{1}{n_i} \sum_{j=1}^{n_i} \sigma_j, \tag{1}$$

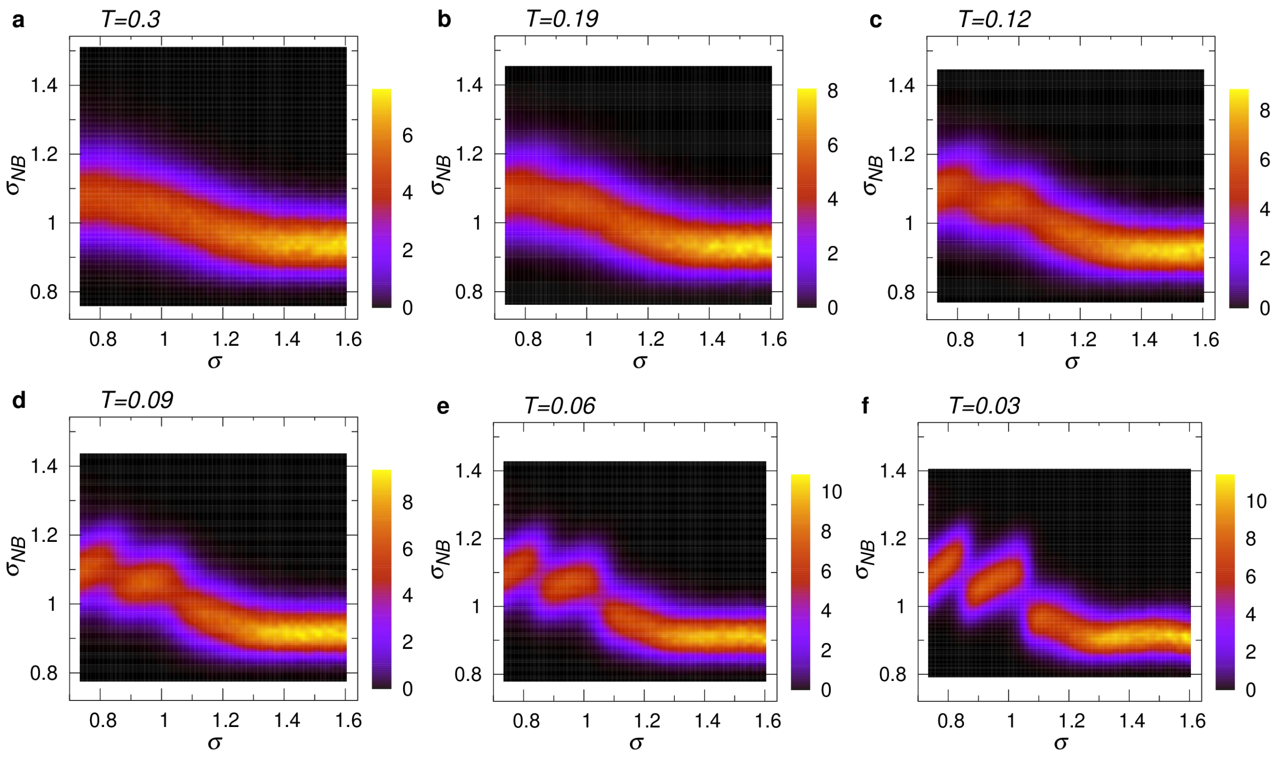

Supplementary Figure 4: **Compositional order over a range of temperatures**. **a-f,** Contour plots of the relation between the neighbour particles' average diameter σ_{NB} and the central particle diameter σ via the conditional probability distribution $P(\tilde{\sigma}|\sigma) = P(\tilde{\sigma}, \sigma)/P(\sigma)$, with the corresponding temperatures indicated by the labels.

where n_i is the number of nearest neighbours of particle i, and the summation goes over all its neighbours. The conditional probability distribution $P(\tilde{\sigma}|\sigma) = P(\tilde{\sigma}, \sigma)/P(\sigma)$ is calculated to see how the particle size of neighbours is coupled with the central particle. Here, $P(\tilde{\sigma}, \sigma)$ is the joint probability distribution of $\tilde{\sigma}$ and σ. Figure 4 shows $P(\tilde{\sigma}|\sigma)$ for a range of temperatures. Interestingly, even at $T = 0.3$, which is much higher than the onset temperature of the system, there is already a clear negative correlation between σ and $\tilde{\sigma}$. This indicates a significant compositional order, i.e., a strong preference between particles with very different sizes even at high temperatures, which is expected to result from the nonadditive interaction. At the crossover temperature of the exotic compositional order, additional features emerge at the low-σ side, which becomes significant at even lower temperatures. At $T = 0.03$, three sections can be observed in $P(\tilde{\sigma}|\sigma)$, reflecting the three major components of the system, i.e., the small, medium, and large particles with $z < 6$, $z = 6$, and $z > 6$. Combined with the results shown in Fig. 3, we expect particles with small and medium sizes to have particular local structures. Therefore, the size ratio between the central and neighbouring particles is mostly constant, leading to the positive correlation in $P(\tilde{\sigma}|\sigma)$. For the largest particles, because of the large number of neighbours and the more irregular local structures, the competition between local ordering and the effect of nonadditivity leads to a slightly winding $P(\tilde{\sigma}|\sigma)$. Such unusual features reflected in $P(\tilde{\sigma}|\sigma)$ are different from that in a model of polydisperse hard spheres with additive interactions [2], indicating that it is not a simple kind of compositional order. This result suggests that the exotic compositional order is a peculiar feature of the model glass former in this study.

SUPPLEMENTARY NOTE 3. ANALYSIS OF DYNAMICS

Here we characterise the structure relaxation using standard molecular dynamics simulations [3]. Figure 5a shows the temperature dependence of self-intermediate scattering functions $F_s(k,t)$ for a range of temperatures. The structural relaxation time τ_α is defined from $F_s(k,\tau_\alpha) = 1/e$. Figure 5b shows the temperature dependence of τ_α, which is fitted by the Vogel-Fulcher-Tammann (VFT) relation $\tau_\alpha = \tau_0 \exp[BT_0/(T - T_0)]$. We find $T_0 = 0.067$, corresponding to the hypothetical ideal glass transition temperature where (physical) τ_α is expected to diverge. Since particles with

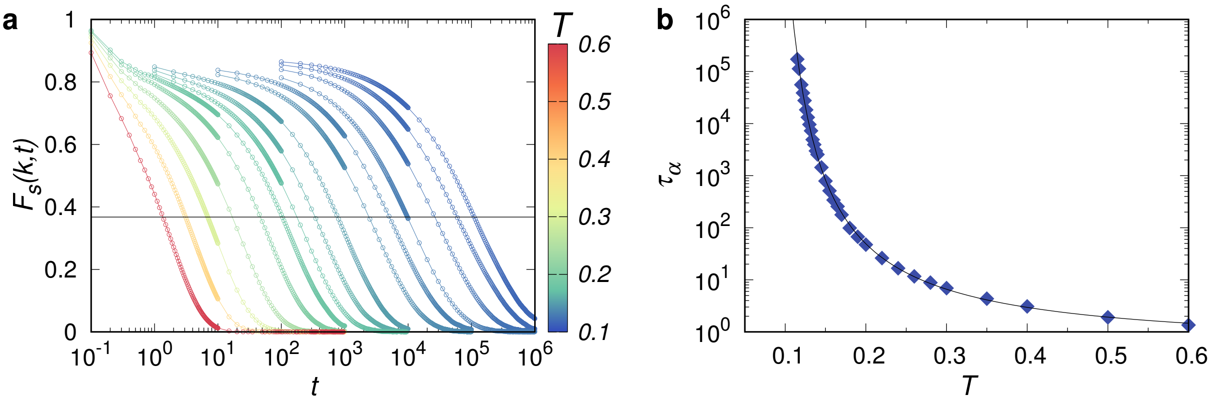

Supplementary Figure 5: **Temperature dependence of structural relaxation**. **a,** Temperature dependence of self-intermediate scattering function, $F_s(k,t)$. The solid line indicates $F_s(k,t) = 1/e$, which defines the structural relaxation time τ_α. **b,** Temperature dependence of τ_α, which is fitted according to the VFT law $\tau_\alpha = \tau_0 \exp[BT_0/(T - T_0)]$, from which we extract the hypothetical ideal glass transition temperature $T_0 = 0.067$.

different sizes play different roles in the exotic compositional order, we also characterise the partial self-intermediate scattering functions for particles with the smallest, medium, and largest sizes (32%, 39%, and 29%, respectively), which are shown in Fig. 6.

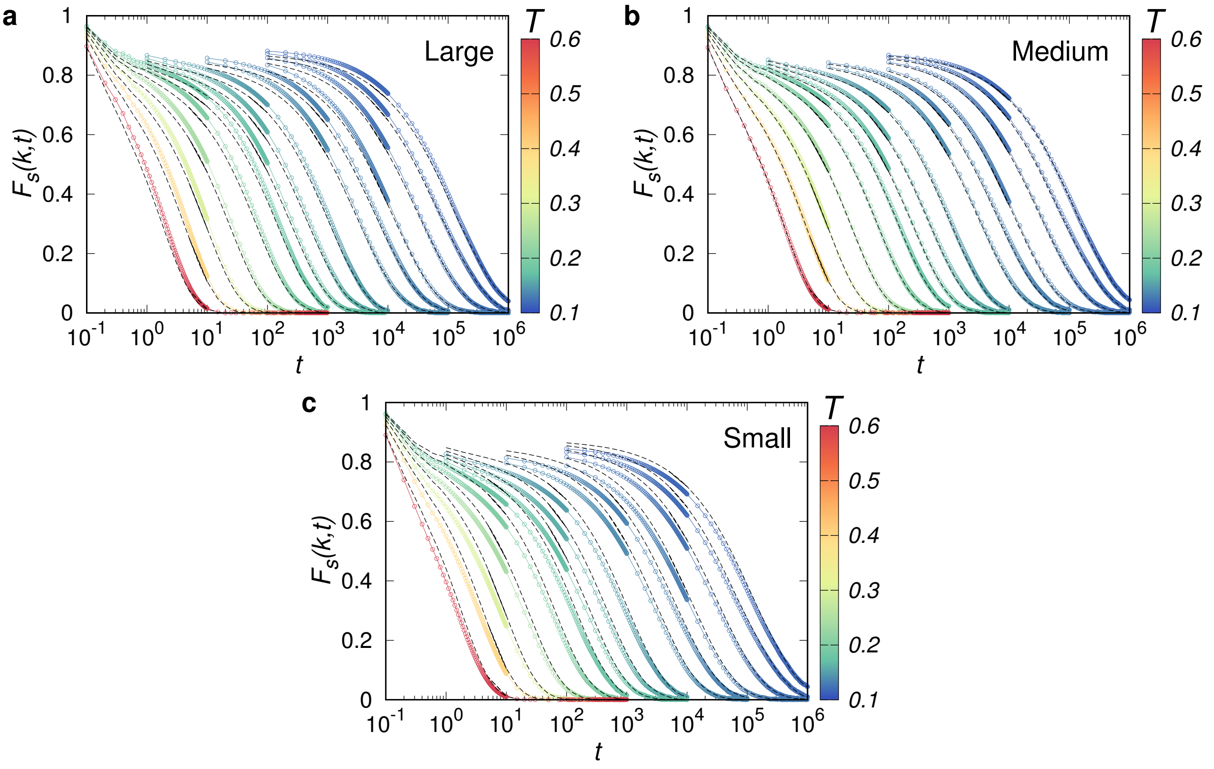

Supplementary Figure 6: **Structural relaxation of particles with different sizes**. **a-c,** Temperature dependence of the partial self-intermediate scattering functions for particles with the smallest, medium, and largest sizes (32%, 39%, and 29%, respectively). As a reference, the corresponding self-intermediate scattering function of the whole system is plotted with dashed curves.

SUPPLEMENTARY REFERENCES

[1] Berthier, L., Charbonneau, P., Ninarello, A., Ozawa, M. & Yaida, S. Zero-temperature glass transition in two dimensions. *Nat. Commun.* **10**, 4875 (2019).
[2] Coslovich, D., Ozawa, M. & Berthier, L. Local order and crystallization of dense polydisperse hard spheres. *J. Phys. Condens. Matter* **30**, 144004 (2018).
[3] Allen, M. P. & Tildesley, D. J. *Computer simulation of liquids* (Oxford university press, 2017).
